# Time-Course Transcriptome Analysis Reveals Resistance Genes of *Panax ginseng* Induced by *Cylindrocarpon destructans* Infection Using RNA-Seq

**DOI:** 10.1371/journal.pone.0149408

**Published:** 2016-02-18

**Authors:** Yuan Gao, Xiaoli He, Bin Wu, Qiliang Long, Tianwei Shao, Zi Wang, Jianhe Wei, Yong Li, Wanlong Ding

**Affiliations:** Institute of Medicinal Plant Development, Chinese Academy of Medical Sciences and Peking Union Medical College, Beijing, China; Chinese Academy of Medical Sciences, Peking Union Medical College, CHINA

## Abstract

*Panax ginseng* C. A. Meyer is a highly valued medicinal plant. *Cylindrocarpon destructans* is a destructive pathogen that causes root rot and significantly reduces the quality and yield of *P*. *ginseng*. However, an efficient method to control root rot remains unavailable because of insufficient understanding of the molecular mechanism underlying *C*. *destructans*-*P*. *ginseng* interaction. In this study, *C*. *destructans*-induced transcriptomes at different time points were investigated using RNA sequencing (RNA-Seq). *De novo* assembly produced 73,335 unigenes for the *P*. *ginseng* transcriptome after *C*. *destructans* infection, in which 3,839 unigenes were up-regulated. Notably, the abundance of the up-regulated unigenes sharply increased at 0.5 d postinoculation to provide effector-triggered immunity. In total, 24 of 26 randomly selected unigenes can be validated using quantitative reverse transcription (qRT)-PCR. Gene ontology enrichment analysis of these unigenes showed that “defense response to fungus”, “defense response” and “response to stress” were enriched. In addition, differentially expressed transcription factors involved in the hormone signaling pathways after *C*. *destructans* infection were identified. Finally, differentially expressed unigenes involved in reactive oxygen species and ginsenoside biosynthetic pathway during *C*. *destructans* infection were indentified. To our knowledge, this study is the first to report on the dynamic transcriptome triggered by *C*. *destructans*. These results improve our understanding of disease resistance in *P*. *ginseng* and provide a useful resource for quick detection of induced markers in *P*. *ginseng* before the comprehensive outbreak of this disease caused by *C*. *destructans*.

## Introduction

*Panax ginseng* C. A. Meyer of the Araliaceae family of perennial plants is a highly valued medicinal plant that is native to East Asian countries such as China, Korea, and Japan [1]. For thousands of years, *P*. *ginseng* root has been widely used as a traditional medicine to promote vitality, enhance physical performance, and increase resistance to stress and aging [2]. However, *P*. *ginseng* production requires a five- to six-year cultivation period, which results in a long exposure to soilborne pathogens. The accumulation of *Cylindrocarpon destructans*, a destructive pathogen present in the ginseng rhizosphere soil, causes serious root rot disease in *P*. *ginseng* and significantly decreases the quality and yield of *P*. *ginseng* [3]. *C*. *destructans* may survive in soil for over 10 years, thereby complicating the control of root rot [4]. The accumulation of soilborne pathogens and allelochemicals in *P*. *ginseng* cultivating fields prevents the continuous cultivation of *P*. *ginseng*. In addition, commercial fungicides exert limited effects on controlling *P*. *ginseng* root rot caused by *C*. *destructans* [3]. Furthermore, effective and environment-friendly biocontrol agents or resistant *P*. *ginseng* cultivars are currently unavailable. Hence, detection of the change of *C*. *destructans*-induced markers at early stage and taking measures in advance was important to ensure the high yield and quality of *P*. *ginseng*.

Previous study shown that *C*. *destructans* can also cause disease symptoms on roots in other species, such as strawberry [5, 6] and grapevines [7, 8]. Comparing with the retarding development in strawberry, *C*. *destructans* may cause severely root rot symptom, and finally resulted in the death of *P*. *ginseng*. Moreover, understanding the molecular mechanism underlying *P*. *ginseng* root rot is necessary to effectively control this disease in the field. Researchers studied the genetic diversity and pathogenicity of *C*. *destructans* [4, 9]. However, the *P*. *ginseng* transcriptome during *C*. *destructans* infection and the molecular mechanism of infection-response underlying *C*. *destructans*-*P*. *ginseng* interaction remain unknown. Gene expression profiling after pathogen infection elucidates plant-pathogen interactions. The current study aims to determine the response of *P*. *ginseng* to *C*. *destructans *infection to reveal defense-related unigenes involved in the plant-pathogen interaction. To achieve this objective, time-course transcriptome libraries were sequenced using Solexa/Illumina RNA-Seq technology to produce high-quality assembly sequences for further quantitative expression analysis. Important differentially expressed transcription factors (TFs) involved in the hormone signaling pathways during disease infection were also identified. Results showed that the unigenes involved in pathogen infection response were dramatically up-regulated at 0.5 d postinoculation (DPI). This study not only revealed pathogen-responsive unigenes for further functional research on accelerating disease-resistant breeding but also provided candidate genes for early detection of induced markers in *P*. *ginseng* to prevent the comprehensive outbreak of root rot disease caused by *C*. *destructans*.

## Materials and Methods

### Plant materials

Roots of three-year-old *P*. *ginseng* cv. Damaya were collected from the experimental field of the Institute of Medicinal Plant Development, Chinese Academy of Medical Sciences. No specific permits were required for the described field studies. The *P*. *ginseng* chosen in this study is susceptible to *C*. *destructans* infection. Healthy *P*. *ginseng* roots were sterilized using 50% carbendazol wettable powder (diluted 800 times) for 10 min and then rinsed thrice in distilled water. The plants were transplanted into sterilized silica sand in plastic square pots (40 cm × 20 cm × 15 cm) with a density of 48 plants/pot and placed in a greenhouse under a 16 h/8 h light/dark cycle at 25°C to control soil moisture, temperature, fertility, pathogens, and other unpredictable factors that may influence disease severity and pathogen growth [10]. Hoagland nutrient solution [11] was used to provide essential nutrients for the normal growth of the *P*. *ginseng* plants.

### Inoculation with *C*. *destructans* and RNA extraction

*C*. *destructans* was inoculated and cultured in liquid potato dextrose medium at 25°C on a shaking incubator for 7 d. Subsequently, spore concentrations were observed with a hemocytometer. After the leaves of the plants fully spread, 250 ml of *C*. *destructans* spore suspension (4.3 × 10^6^ condia·ml^−1^) was sprayed to each pot of sterilized silica sand so that *C*. *destructans* could be adsorbed by roots of *P*. *ginseng* evenly. The polysaccharide content in the main root of *P*. *ginseng* was extremely high, and fibrous roots of *P*. *ginseng* have stronger response to pathogen infection than the main root. Thus, fibrous roots of *P*. *ginseng* from three plants were collected and pooled for RNA extraction to ensure the quality for high-throughput sequencing. Time-course disease responses were investigated at 0.25, 0.5, 1, 4, 7, and 12 DPI. The fibrous roots harvested from the uninfected *P*. *ginseng* plants served as controls (0 DPI). The fibrous roots of three plants at each time point were rinsed with distilled water and then pooled. The roots were briefly blotted with several pieces of dry filter paper and then immediately frozen in liquid nitrogen until RNA extraction. The total RNA was extracted using TRIzol Reagent in accordance with the manufacturer’s protocols and then treated with RNase-free DNase to eliminate genomic DNA contamination. The total RNA for RNA-Seq library construction was quantified on the Thermo Scientific NanoDrop 2000 spectrophotometer (Wilmington, DE) and the Agilent 2100 Bioanalyzer (Santa Clara, CA).

### Library construction and sequencing

Transcriptome libraries were constructed using the Illumina kit in accordance with the manufacturer’s instructions. In brief, the total RNA treated with DNase was purified and isolated using Magnetic Oligo (dT) beads. Then, the purified RNA was sheared to approximately 330 nt fragments prior to cDNA synthesis. Short fragments were purified and ligated to sequencing adapters. Fragments with suitable sizes (400–500 nt) on the basis of agarose gel electrophoresis were selected as templates for PCR amplification in order to isolated and purify the cDNA fragments for sequencing. The final PCR products were sequenced using Illumina HiSeq™ 2000 as 100 nt paired-end (PE) Illumina reads. The quality of raw RNA-Seq reads was filtered using the following criteria: (1) reads including adapter sequencing or empty adapter were filtered; (2) reads for which Ns comprised more than 3% of the total length were discarded; and (3) reads including low-quality bases that comprise more than 15% of the total reads were discarded.

### Transcriptome assembly and annotation

The RNA-Seq reads were subjected to *de novo* transcriptome assembly by using Trinity assembly software [12] to obtain high-quality transcript sequences. Large RNA-Seq datasets were normalized using the Trinity in silico normalization utility. Then, PE RNA-Seq reads with default parameters were subjected to *de novo* transcriptome assembly by using Trinity (version: r20140413p1). Transdecoder with the default parameters was used to identify open reading frames [13]. Then, the assembled sequences were used for a homology search against the UniProt databases by ncbi-blast-2.2.27+ with an E-value of 10^−6^. Gene ontology (GO) terms were assigned to assemble the sequences on the basis of the UniProt databases (EMBL Uniprot eggNOG/GO pathways databases). GO terms were analyzed to classify the unigenes into biological process, cellular component, and molecular function. GO enrichment analysis (*P* < 0.05; hypergeometric test with Benjamini and Hochberg false discovery rate correction) was performed on selected unigenes using BINGO 3.0.2 [14] in accordance with the custom GO annotation files to identify the enriched GO terms.

### Identification of *C*. *destructans* sequences

*C*. *destructans* EST sequences were downloaded from the NCBI EST database. Assembly sequences of *C*. *destructans* were identified using ncbi-blast-2.2.27+ with more than 90% identity and E-value less than 0.00001.

### Gene expression analysis

Bowtie2 2.2.3 [12] was used to map the RNA-Seq reads with the assembled sequences to calculate the read counts for each unigene. Only PE reads paired with unique locations were retained to accurately calculate the expression values of the assembled unigenes. The transcript levels of each unigene were measured and normalized as the fragments per kilobase of exon per million fragments mapped (FPKM), which is analogous to reads per kilobase of exon model per million mapped reads [15]. Unigenes with low expression have questionable biological significance and are difficult to validate. Therefore, in this study, the minimum expression threshold for FPKM > 3 was observed in at least one library. The *P* value was assessed using an MA-plot-based method with the random sampling model in the statistical R package DEGseq [16]. Then, differentially expressed genes (DEGs) were filtered at a fold change > 3 and a *P* < 0.001 (Benjamini and Hochberg false discovery rate correction) as the threshold by performing pair-wise comparisons of infected samples with noninfected samples. The clustering algorithm was used to analyze time-course gene expression data [17].

### Validation of DEGs and defense-related genes through quantitative reverse transcription (qRT) PCR

RNA for qRT-PCR validation was extracted from fibrous roots of *P*. *ginseng* as a different set of biological samples under the same condition as that used in RNA-Seq. The extracted RNA was digested with RNase-free DNase I (Promega) to remove genomic DNA contamination. Reverse transcription was performed using 1 μg of total RNA for each example and 200 U M-MLV Transcriptase (TaKaRa) in a 10 μl volume. The reaction was conducted at 70°C for 10 min, 42°C for 60 min, and 70°C for 15 min. The resulting cDNA was diluted to 800 μl with sterile water. qPCR was conducted using the BIO-RAD CFX system (BIO-RAD) in triplicate. Gene-specific primers were designed using Primer3 (http://bioinfo.ut.ee/primer3/). The primers used in this study are listed in S1 Table. An eEF-1α gene (c64517_g1) was selected as the endogenous control because of its relatively stable mRNA across all the time points. PCR was conducted in a 20 μl volume containing 4 μl of diluted cDNA, 250 nM forward primer, 250 nM reverse primer, and 1 × SYBR Premix Ex Taq II (TaKaRa) under the following conditions: 95°C for 3 min and then 40 cycles of 95°C for 15 s, 59°C for 15 s, and 72°C for 15 s. Melting curve analyses were performed to verify the specificity of the Bio-Rad CFX Manager software. Relative expression levels were calculated using the 2^-ΔΔCT^ method [18].

### Identification of disease RGs

To identify RGs in *P*. *ginseng*, manually curated reference RGs were downloaded from the Plant Resistance Gene Database (PRGdb, http://prgdb.crg.eu/). BLAST with a stringent E-value cutoff of 10^−15^ was used to identify homologous sequences in the *P*. *ginseng* assembly transcriptome.

## Results

### Sequencing and *de novo* assembly of the *P*. *ginseng* transcriptome

The innate immune responses of *P*. *ginseng* to *C*. *destructans* infection were explored by constructing RNA-Seq libraries for 0, 0.25, 0.5, 1, 4, 7, and 12 DPI to maximize the transcript diversity and by sequencing these libraries using the Illumina/Solexa high-throughput sequencing platform (Table 1). After removing low-quality reads, 27, 27, 28, 26, 23, 27, and 29 million 100 bp PE clean reads were generated from one control and six treatments (Table 1). More than 180 million 100 nt PE reads were ultimately generated. All of the sequencing reads are available in the Short Read Archive database under Accession Number SRR1639601.

**Table 1:** Summary of Illumina sequencing and transcriptome assemblies for RNA-Seq libraries.

Sample	Reads (PE)	Length	Total bases (×100bp)	GC%^a^	Mapping (ratio)	Unique	FPKM >3
0.25 DPI	27,086,424×2	100,100	54,172,848	43,43	45,396,150 (84%)	18,770,088×2	20,298
0.5 DPI	27,961,332×2	100,100	55,922,664	43,43	47,263,043 (85%)	19,721,325×2	19,385
1 DPI	26,009,438×2	100,100	52,018,876	43,43	44,250,820 (85%)	18,602,589×2	19,789
4 DPI	23,420,903×2	100,100	46,841,806	43,43	39,345,176 (84%)	16,267,657×2	20,543
7 DPI	27,215,622×2	100,100	54,431,244	43,44	45,964,650 (84%)	20,190,441×2	20,104
12 DPI	28,951,315×2	100,100	57,902,630	43,43	47,795,838 (83%)	19,968,169×2	21,123

The *P*. *ginseng* plant genome sequence is currently unavailable; therefore, *de novo* transcriptome assembly was adopted before calculating the expression level. PE reads from different libraries were assembled into transcript sequences by using Trinity [13]. *De novo* assembly yielded 73,335 unigenes for the *P*. *ginseng* transcriptome after *C*. *destructans* infection. The average length for the assembled sequence was 1,385 bp, suggesting that the quality of the assembly was high enough to conduct downstream analyses. We blasted the Trinity assembly with *C*. *destructans* EST sequences and found only 10 assembly sequences that were similar to *C*. *destructans* sequences. We excluded this small amount of *C*. *destructans* sequences before the downstream analysis.

### Gene expression analysis

**Fig 1:**
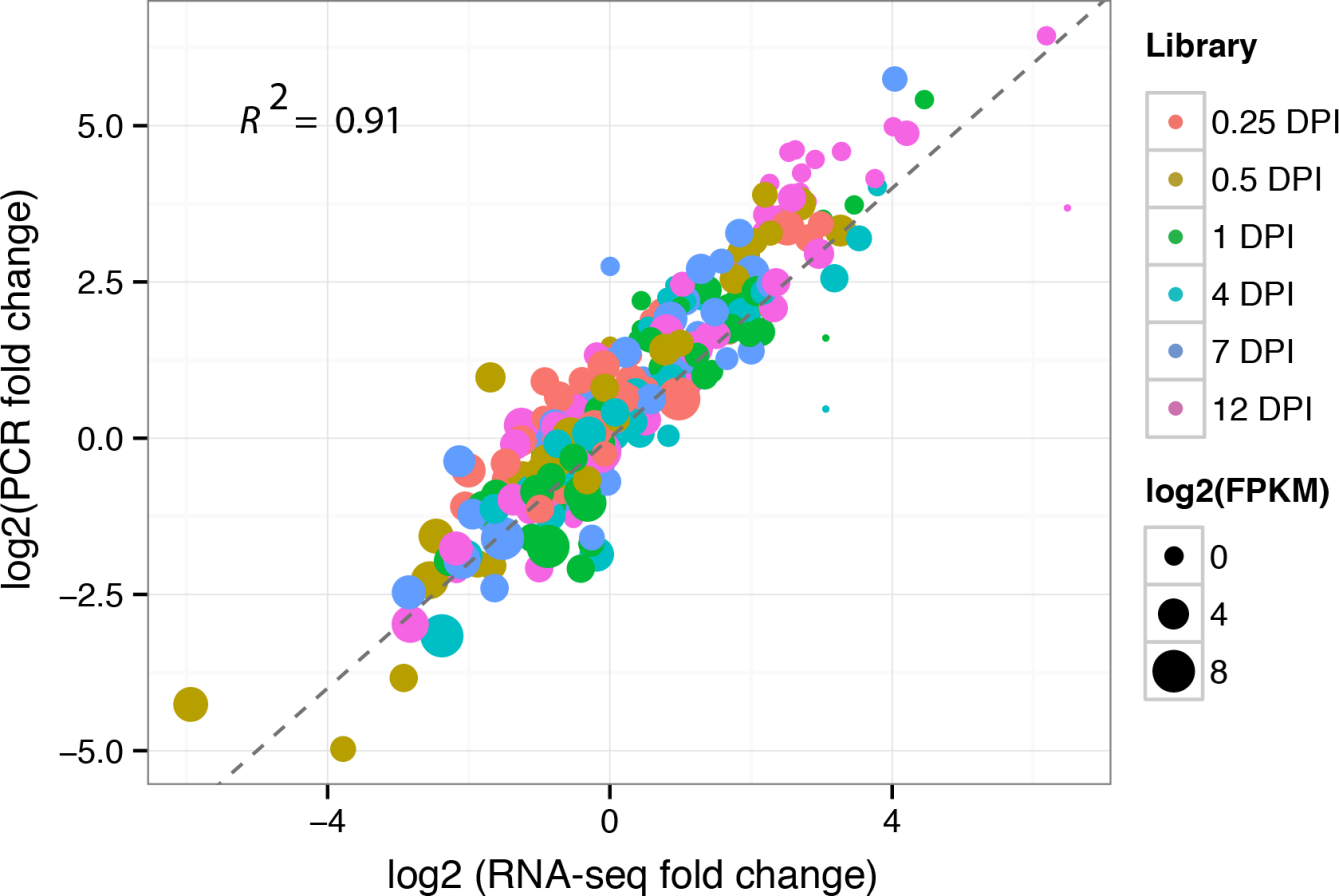
Correlation between qRT-PCR and RNA-Seq. The X-axis represents the log2 fold change of RNA-Seq. The Y-axis indicates the log2 value of fold change from qRT-PCR. Different colors represent different time points. The size of each point is proportional to the log2 (FPKM) in 0 DPI.

**Fig 2:**
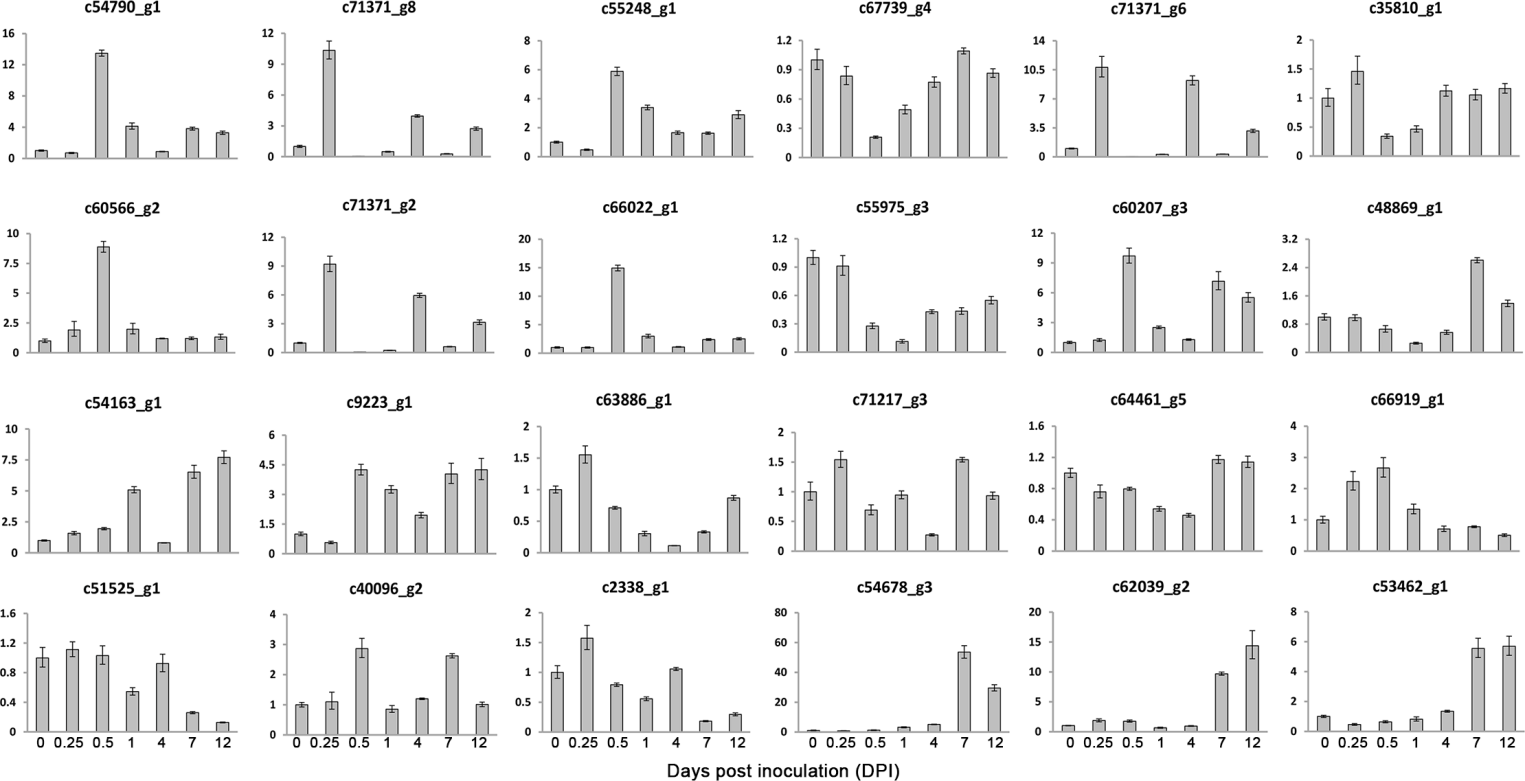
Validation of 24 randomly selected genes through qRT-PCR. Fold changes of the transcript levels at different time points are shown. The average expression level at 0 DPI was set to 1. Error bars represent standard error from three independent experimental replicates.

Approximately 84% of the reads were successfully mapped to the assembled transcript sequences by using Bowtie2 [12] alignment (Table 1). The ratio of matched reads was not significantly different. A total of 46,616,140, 37,540,176, 39,442,650, 37,205,178, 32,535,314, 40,380,882, and 39,936,338 reads were mapped in pairs with unique locations for the libraries of 0, 0.25, 0.5, 1, 4, 7, and 12 DPI, respectively (Table 1). Gene expression levels were measured and normalized as FPKM to quantify the expression of each unigene [15]. Only PE reads paired with a unique location were retained to accurately calculate the expression values of the assembled unigenes. The unigenes with high expression values included anti-sense ribosomal RNA transcript protein 2 (c71357_g1), phloem protein 2-like A4 (c59751_g4), omega-6 fatty acid desaturase (c69775_g1), auxin-repressed 12.5 kD protein (c50590_g1), ribonuclease 2 (c61159_g1), and heat shock cognate protein 80 (c60763_g1). Ribonuclease 2 [19] and shock cognate protein 80 [20] responded to biotic stimuli and stress, respectively. In total, 19524, 20298, 19385, 19789, 20543, 20104, and 21123 unigenes were expressed in the libraries of 0.25, 0.5, 1, 4, 7, and 12 DPI, respectively (Table 1), with corresponding average FPKMs of 27, 28, 27, 27, 28, and 26. We randomly selected 26 unigenes and performed qRT-PCR analysis with specific primers at seven time points to validate the reliability of the RNA-Seq results. In order to further validate the reliability of our RNA-seq result, we also selected 24 unigenes that shown differential expression in reactive oxygen species (ROS) and ginsenoside biosynthetic pathway. At the same time, six defense response genes were also selected for validation. In total, we validated 56 genes in this study by qRT-PCR. The correlation coefficient for these unigenes was 0.91, which indicating a high correlation between qRT-PCR and RNA-Seq results (Fig 1). The experiment validation based on qRT-PCR suggested that the results of RNA-Seq were solid. The expression patterns of the 24 randomly selected unigenes (92%) identified by RNA-Seq can be validated using qRT-PCR (Fig 2) and the expression trends of RNA-Seq were shown at S1 Fig. The validation results by qRT-PCR for 24 DEGs from ROS and ginsenoside biosynthetic pathway were presented at S2 Fig.

### Cluster of time-course RNA-Seq data

**Fig 3:**
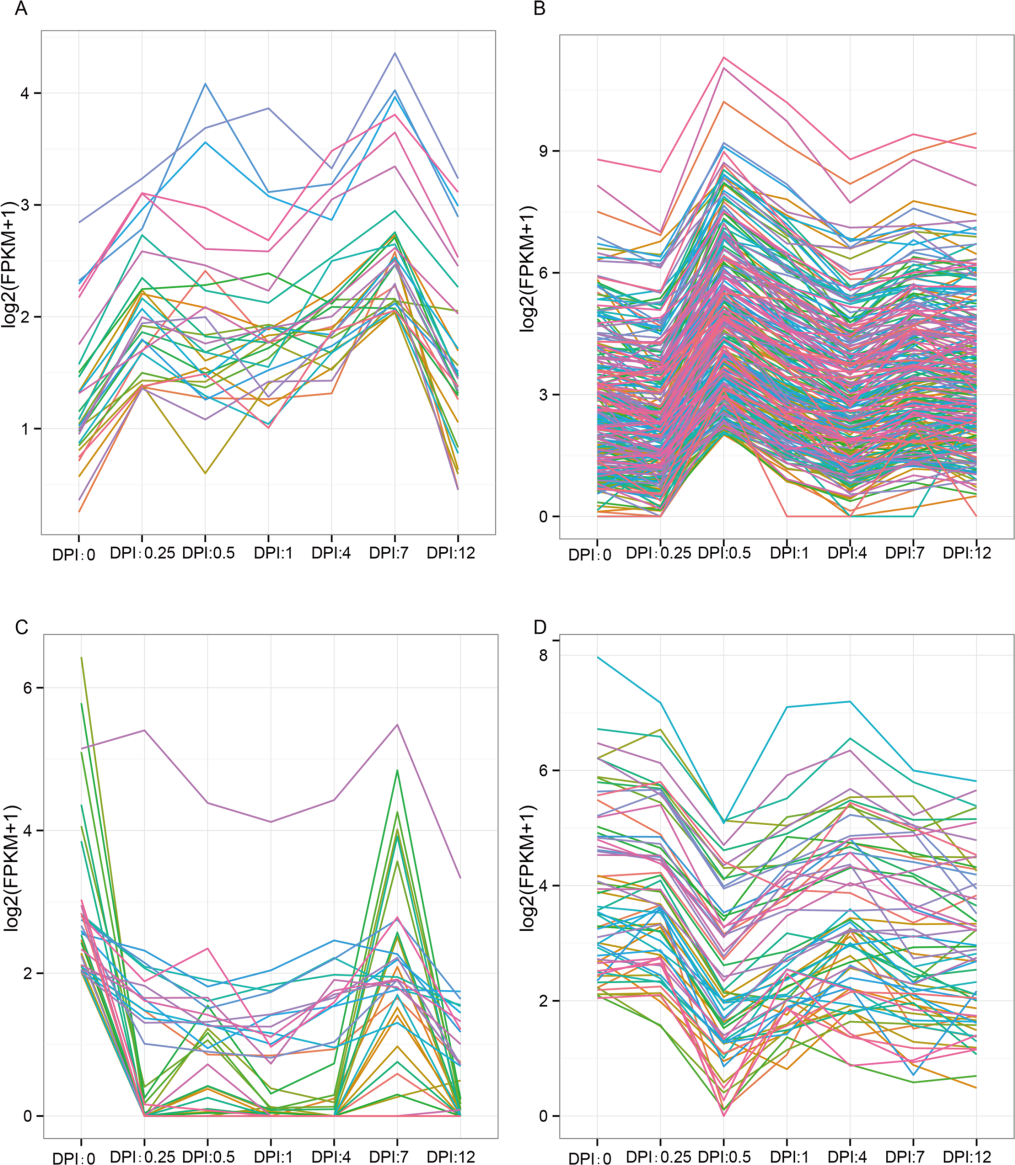
Most represented patterns of time-course data for DEGs. Unigene expression profiles are clustered using the Short Time-series Expression Minor program. The y-axes represent expression level and the x-axes represent different stages after *C*. *destructans* infection. A) Expression increased at 0.25 DPI relative to the 0 DPI. In the middle stage, the expression of part genes decreased and then slightly increased at 7 DPI. B) The expression pattern of most of the unigenes rapidly increased at 0.5 DPI relative to the 0 and 0.25 DPI, and then decreased at 4 DPI. At 7 DPI there were slightly increased. C) The expression of part down-regulated unigenes decreased at 0.25 DPI and then remained at low level from 0.5 DPI to 4 DPI. At last two stages the unigenes sharply increased at 7 DPI and sharply decreased at 12 DPI. D) The expression of the down-regulated unigenes sharply decreased at 0.5 DPI relative to the 0 DPI, then other stage maintained relative stable expression.

Normalized FPKMs were provided as input for Short Time-series Expression Miner analysis [17]. An expression matrix was constructed, and the groups of unigenes that varied in concert over different time points were revealed. Unigenes sharing similar expression patterns were clustered, and the most statistically significant profiles were identified (Fig 3). The expression level of some of the unigenes increased at 0.25 DPI, decreased between 0.5 DPI and 4 DPI, and then slightly increased at 7 DPI (Fig 3A). In addition, the expression pattern of most of the up-regulated unigenes rapidly increased at 0.5 DPI (Fig 3B). Notably, the abundance of the up-regulated unigenes sharply increased at 0.5 DPI. The expression level of some of the down-regulated unigenes decreased at 0.25 DPI and then increased at 7 DPI (Fig 3C). The expression profile of most of the down-regulated unigenes sharply decreased at 0.5 DPI (Fig 3D). Considerable expression changes were observed at 0.5 DPI, suggesting that the unigenes related to defense response to fungus served important functions at this time point.

### DEG identification and GO functional enrichment analysis for DEGs

**Fig 4:**
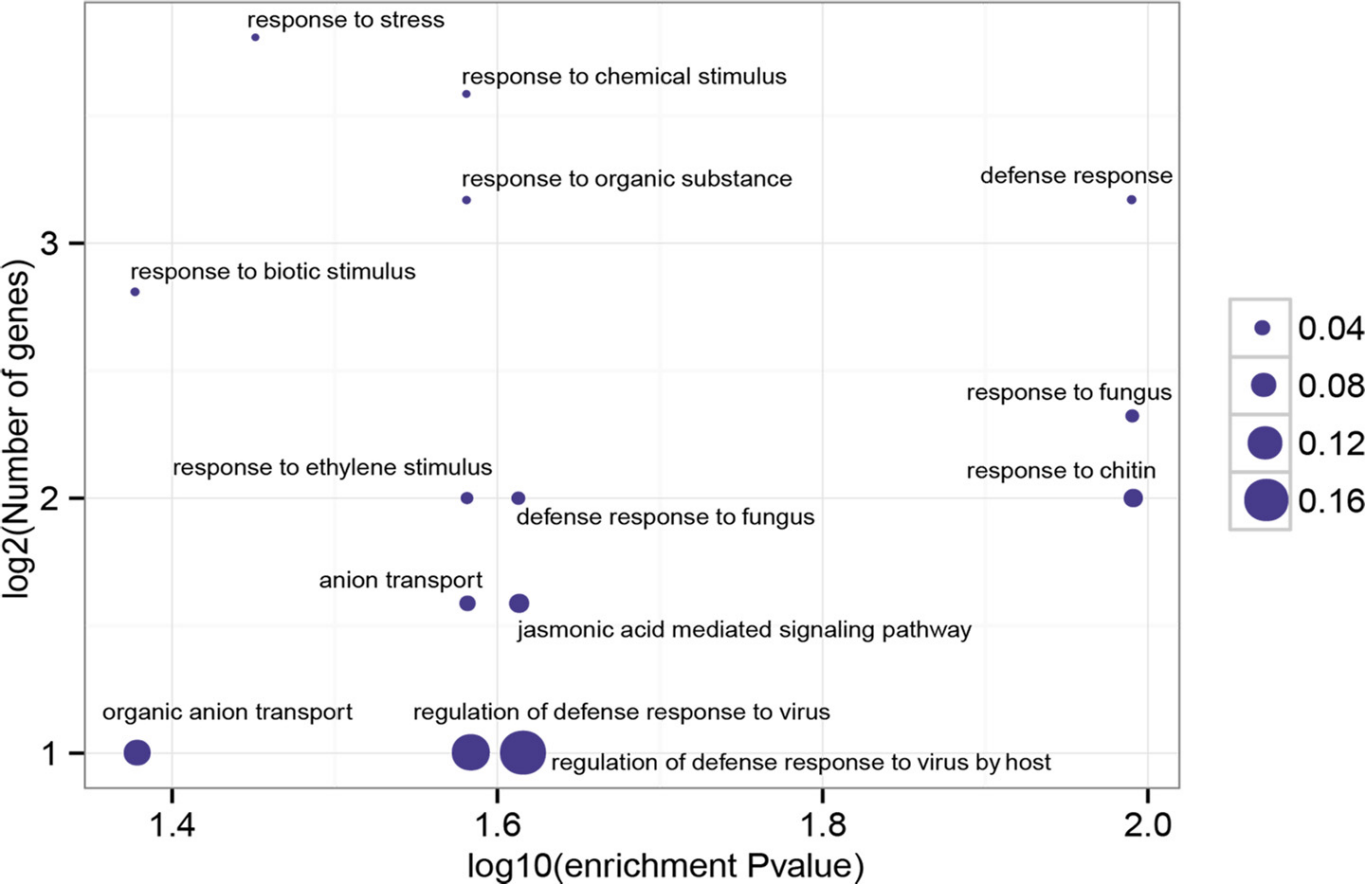
Significant enrichment functional groups of DEGs. The most significant GO terms (*P* < 0.05) of differentially expressed unigenes are graphically presented by comparing with total transcripts. The x-axis represents log10 of the enrichment *P* value. The y-axis indicates the number of unigenes in log2 value. The size of each point is proportional to the percentage (DEG associated with GO terms/All unigenes associated with GO terms).

The ultimate goal of this study is to characterize the dynamic changes in the *P*. *ginseng* transcriptome after *C*. *destructans* infection. Thus, the DEGs in *P*. *ginseng* plants infected by *C*. *destructans* were investigated. To identify significant DEGs, a cutoff of at least a three-fold change and *P* < 0.001 was used. In total, 81, 538, 392, 513, 606, and 2,845 up-regulated unigenes as well as 99, 201, 76, 69, 191, and 280 down-regulated unigenes were identified from the libraries of 0.25, 0.5, 1, 4, 7, and 12 DPI, respectively (Table 2). GO analysis for up-regulated unigenes showed that “defense response to fungus” was enriched (*P* = 1.0250E-2). All of the significant GO terms for DEGs were shown in Fig 4. Enrichment of “jasmonic acid mediated signaling pathway” was correlated well with the previous report that jasmonic acid (JA)-responsive genes are involved in defense response [21]. In total there were seven up-regulated unigenes (c53321_g1, c53833_g1, c55218_g1, c56156_g1, c57783_g2, c60188_g4, c71320_g4) that were involved in the jasmonic acid mediated signaling pathway. The expression of these seven JA related genes increased at 0.5 DPI, suggesting that these genes were induced to obtain systemic resistance at this time point by JA-mediated signaling pathway. “Response to ethylene stimulus” was also enriched in the up-regulated genes. In total there were 13 ethylene related genes identified in the up-regulated genes. These genes were involved in ethylene binding (c67746_g1), ethylene biosynthetic process (c67590_g1 and c59280_g1), ethylene-activated signaling pathway (c53833_g1, c57684_g3, c60188_g4, c61349_g2, c62289_g1, c62289_g2 and c62382_g3), negative regulation of ethylene-activated signaling pathway (c67746_g1), response to ethylene (c54969_g1 and c55218_g1) and ethylene-dependent systemic resistance (c62382_g3 and c67174_g2). It will be interesting to investigate the unigenes with ethylene-dependent systemic resistance in future. The enriched GO terms for the down-regulated unigenes differed from those for the up-regulated unigenes. GO terms associated with “photosynthesis” “carbon fixation”, “photosystem II stabilization”, “red light signaling pathway” and “paclitaxel biosynthetic process” were enriched for the down-regulated unigenes. The above results suggested that the root rot pathogen *C*. *destructans* affected the photosynthesis of the host plant *P*. *ginseng*. Consistently, a previous study reported that leaves infected by foliar pathogens exhibit reduced photosynthetic activity [22]. All identified differentially expressed unigenes with GO annotation were listed in S2 Table. These results provided a comprehensive overview of gene expression and valuable information for further investigation.

**Table 2:** The number of up- and down-regulated DEGs based on pair-wise comparison with control (fold change > 3; *P* < 0.001).

Sample	Up-regulated	Down-regulated
**0.25 DPI**	**81**	**99**
**0.5 DPI**	**538**	**201**
**1 DPI**	**392**	**76**
**4 DPI**	**513**	**69**
**7 DPI**	**606**	**191**
**12 DPI**	**2,845**	**280**

**Fig 5:**
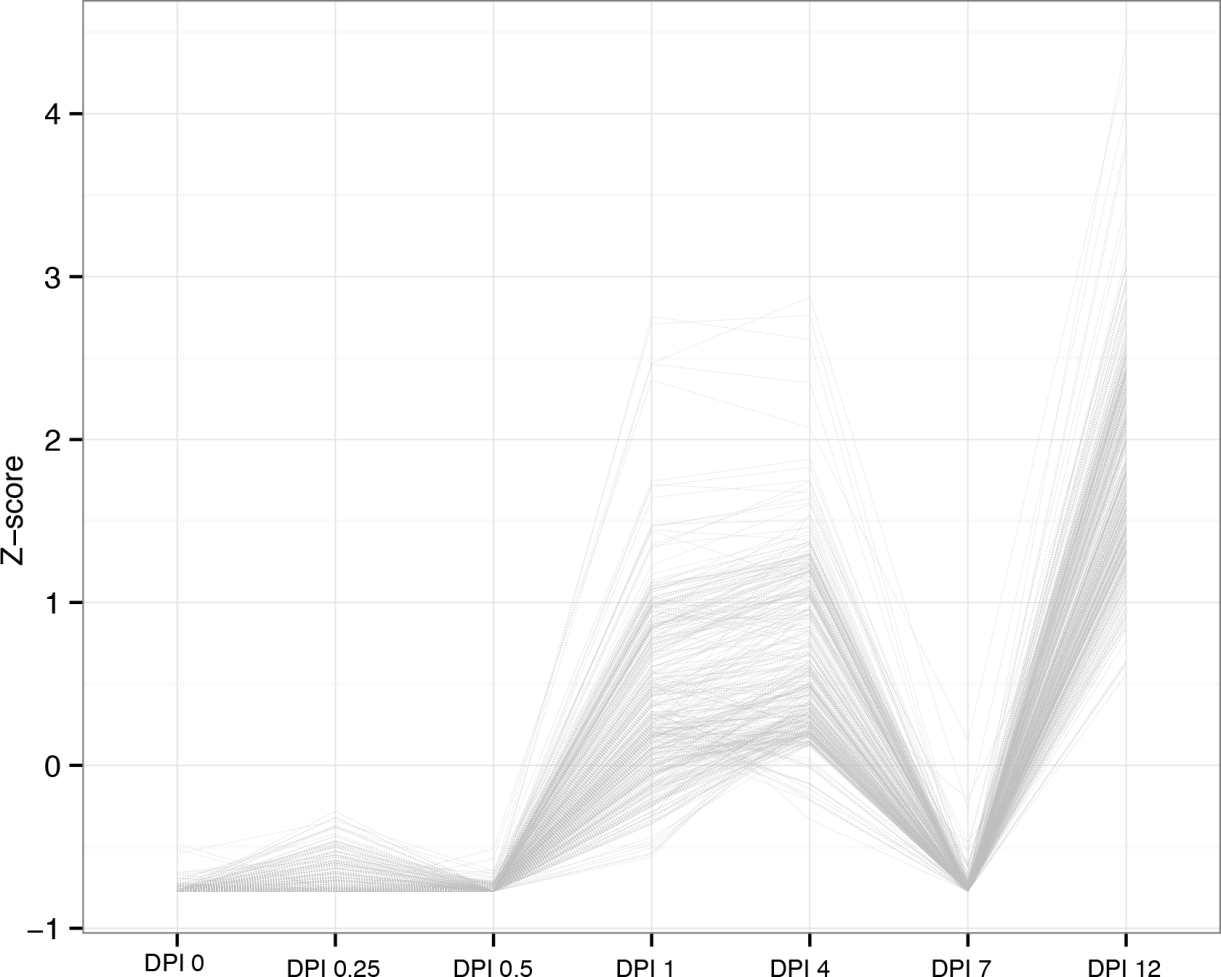
Most represented patterns of time-course data for other comparisions. The y-axes represent Z-score and the x-axes represent different stages after *C*. *destructans* infection.

In order to reveal the dynamic change along the development of infection, we added all the possible combinations and comprising analysis among all the different time points. In total, there were 2,494 up-regulated and 4,526 down-regulated genes (Table 3), respectively. Among these DEGs from new comparisons, we identified one expression patterns that slightly increased at 0.25 DPI, and then presented low expression at 0.5 DPI and 7 DPI (Fig 5). Notably, the abundance of the DEGs sharply increased at 1 DPI, 4 DPI and 12 DPI, respectively. The enriched GO terms for these unigenes associated with “cell cycle process”, “translation” and “transmembrane transport”, which suggested that *C*. *destructans* affected the cell cycle and protein translation of the host plant *P*. *ginseng* at middle and later stage.

**Table 3:** Summary of comparisions among all the time points (fold change > 3; *P* < 0.001).

Pair-wise Comparision	Up	Down
**0.25 vs. 0.5**	**643**	**256**
**0.25 vs. 1**	**548**	**172**
**0.25 vs. 4**	**571**	**92**
**0.25 vs. 7**	**645**	**316**
**0.25 vs. 12**	**3,002**	**509**
**0.5 vs. 1**	**352**	**114**
**0.5 vs. 4**	**624**	**416**
**0.5 vs. 7**	**725**	**494**
**0.5 vs. 12**	**2,808**	**584**
**1 vs. 4**	**252**	**170**
**1 vs. 7**	**530**	**460**
**1 vs. 12**	**2,734**	**292**
**4 vs. 7**	**465**	**610**
**4 vs. 12**	**2,616**	**249**
**7 vs. 12**	**2,436**	**492**

### Defense response analyses

**Fig 6:**
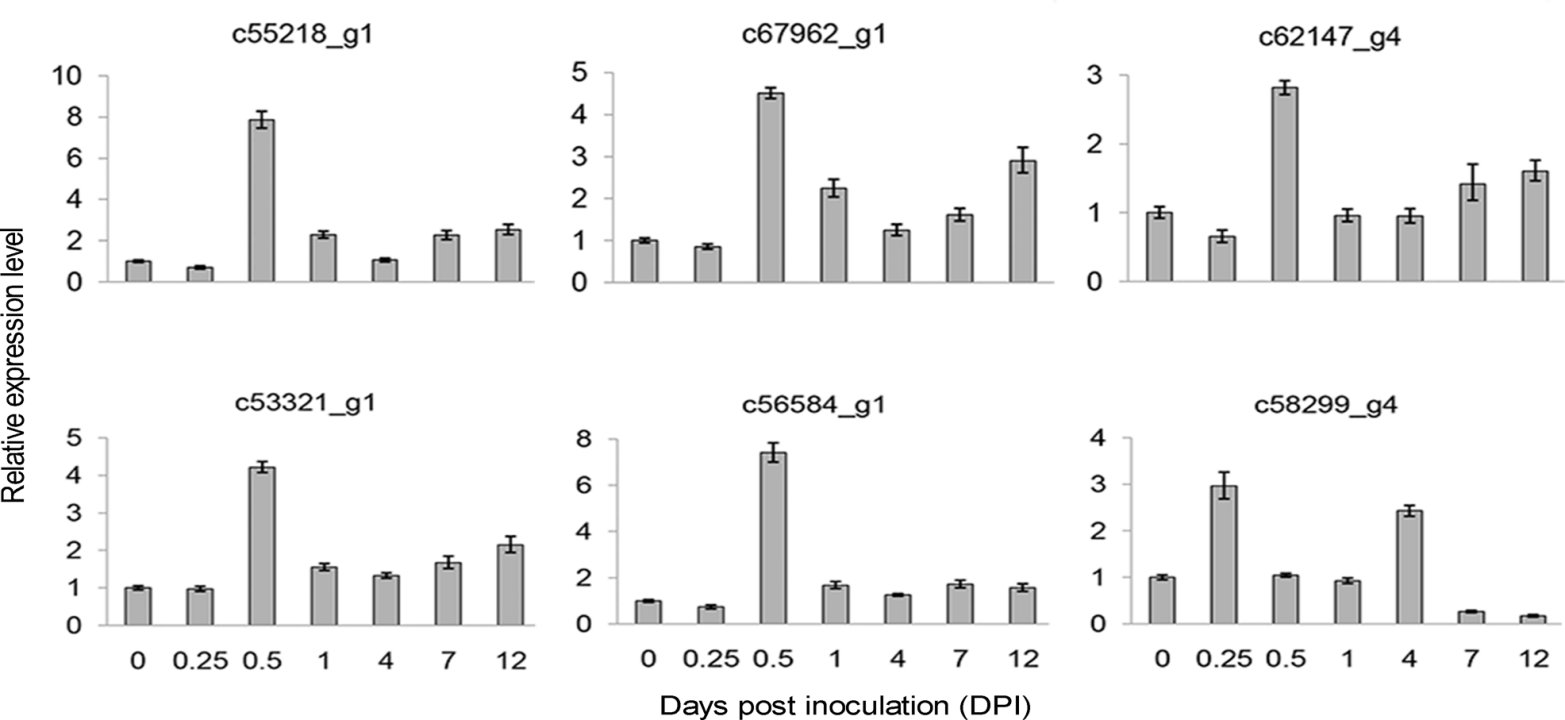
Expression profiles of six randomly selected RGs through qRT-PCR. Fold changes of the transcript levels at different time points are shown. The average expression level at 0 DPI was set to 1. Error bars represent standard error.

Unigenes involved in defense response or pathogenesis (GO: 0006952 and GO: 0009405) were further analyzed. In total, 257 defense response unigenes (DRG) were identified, of which 29 (11% of the total) showed differential expression. The expression profiles of six representative DRGs were validated at different time points by using qRT-PCR (Fig 6). As shown in S3 Fig, qRT-PCR and RNA-Seq analyses revealed consistent expression trends of the DRGs. TFs are important components involved in the interaction between plants and microbes as well as in the regulation of defense-related genes [23]. Among the 29 DEGs, TFs ethylene-responsive transcription factor 2 (c60188_g4), TIFY 10A (c53321_g1), TIFY 10B (c69797_g3), and MYB108 (c55218_g1 and c54969_g1) increased at 0.5 DPI compared with the control. JA is the primary signaling molecule that regulates pathogen resistance in plants [24]. Genes related to the JA pathway are induced by pathogen attack [25]. TIFY 10A and TIFY 10B respond to the JA-mediated signaling pathway [26–28]. These results indicated the existence of a crosstalk for DEGs in response to resistance and hormone in *P*. *ginseng*. Pathogenesis-related protein (PRP) is important in the defense mechanism of *P*. *ginseng* and is up-regulated under biotic stress [29]. In the present study, two PRP unigenes (c55244_g1 and c58299_g4) were differentially expressed after *C*. *destructans* infection (S4 Fig).

### RG expression profiling

**Fig 7:**
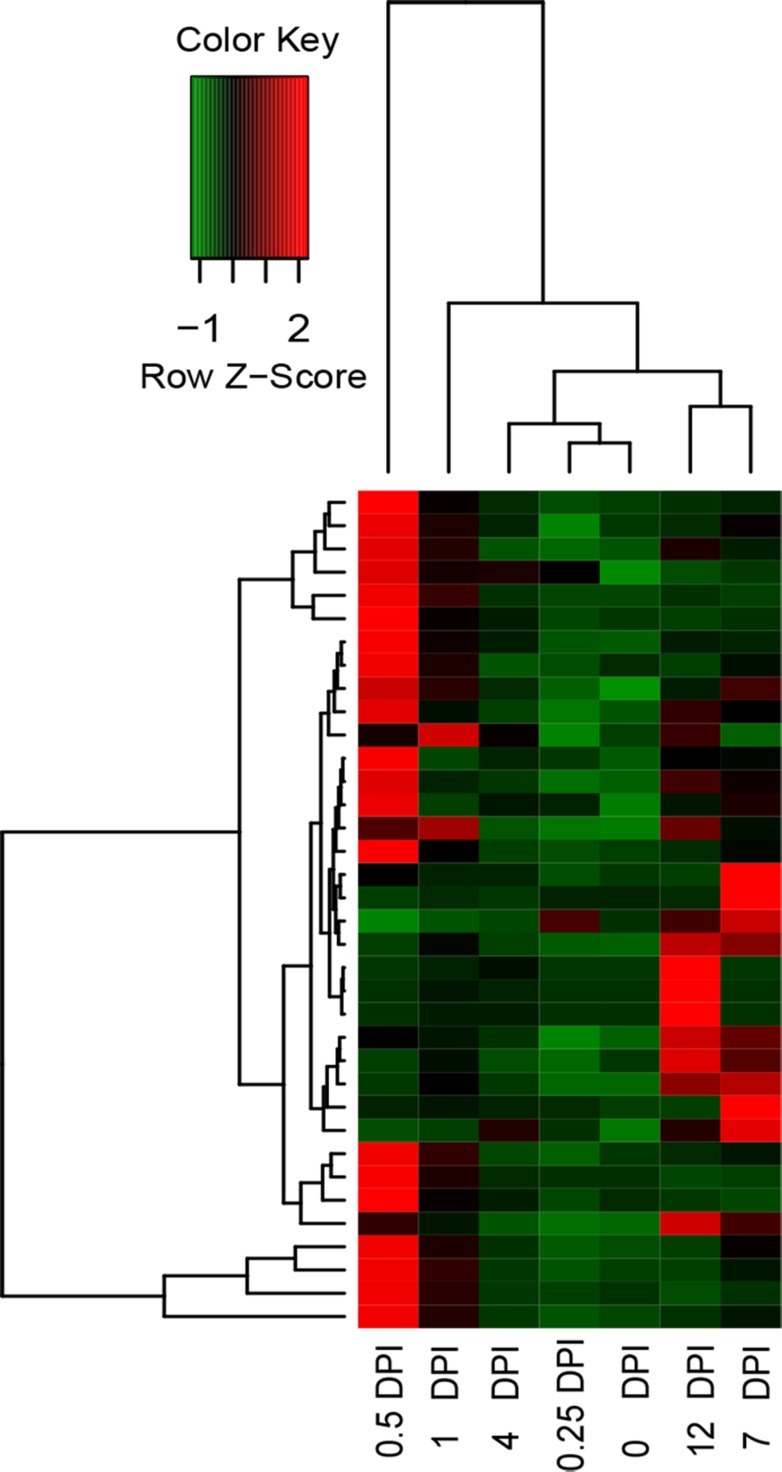
Heatmap showing the FPKM values of up-regulated RGs. The FPKM for the RGs in the control and six infected stages was used for hierarchical analysis. The heatmap shows the expression abundance of the RGs. The colors correspond to the value of FPKM, ranging from green (low expression) to red (high expression).

Basing on the PRGdb database, we matched 1040 unigenes in *P*. *ginseng* with the reference RGs. The low expression levels of the 36 RGs in the control increased (fold change > 3) after *C*. *destructans* infection in at least one DPI library (S5 Fig). In addition, 0.5 DPI was the time point at which *P*. *ginseng* response to *C*. *destructans* infection was dramatically induced (Fig 7). All identified RGs with FPKM and annotation are listed in S3 Table.

### Identification of uniquely expressed unigenes upon *C*. *destructans* infection

**Fig 8:**
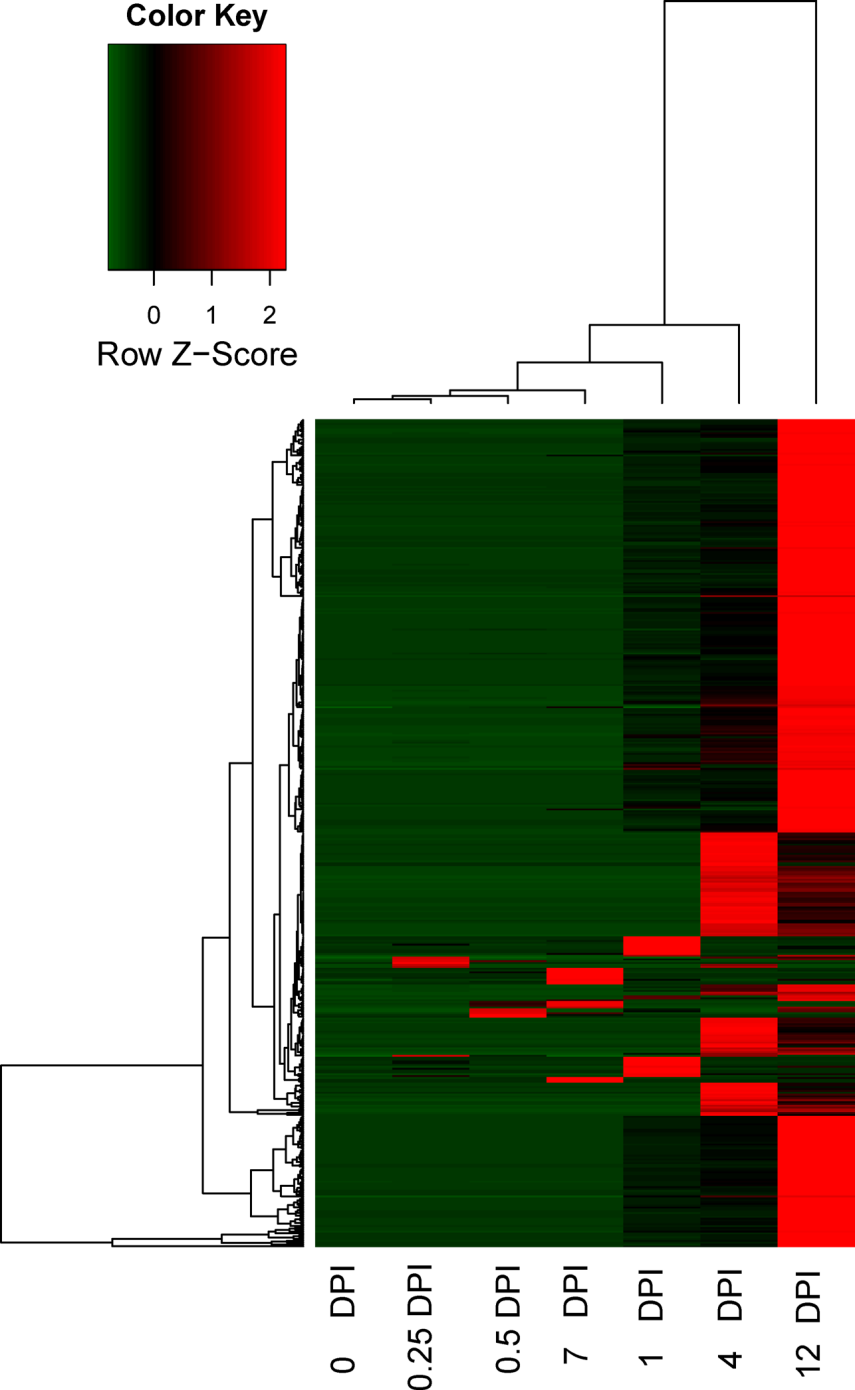
Heatmap showing the FPKM values of uniquely expressed unigenes upon infection. The FPKM for the uniquely expressed unigenes upon infection in the control and six infected stages was used for hierarchical analysis. The colors correspond to the value of FPKM, ranging from green (low expression) to red (high expression).

We indentified uniquely expressed unigenes during early infection by using following cuttoff: (1) the expression of candidate unigenes could not be detected in the uninfected 0 DPI. (2) Among infected library, there was at least one library with RPKM more than 3. By using this cuttoff, we identified 447 unigenes with uniquely expressed. The induced expression of these candidate unigenes has bias at the late time point upon infection (Fig 8). It will be interesting to investigate the candidates in future since uniquely expressed unigenes upon infection were important for systemic resistance.

### Reactive oxygen species and ginsenoside biosynthetic pathway during *C*. *destructans* infection

Plants can produce reactive oxygen species (ROS) that causes oxidative damage during biotic and abiotic stress [30]. The investigation of the ROS can help us to understand the possible function of ROS upon pathogenic infection. In total, we identified 36 ROS-related genes (GO: 0000302 and GO: 0072593), which could response to reactive oxygen species. Among these unigenes, 1 (c61102_g3), 1 (c54370_g1), 2 (c54370_g1, c57939_g2), 7 (c40714_g1, c50821_g1, c52377_g1, c54370_g1, c55185_g1, c55776_g1, c61102_g3) were differentially expressed, which were up-regulated in the libraries of 0.5 DPI, 1 DPI, 4 DPI and 12 DPI, respectively. The qRT-PCR validations for these DEGs could be found at S2 Fig. The RNA-Seq plots were shown at S6 Fig. Among these DEGs, c54370_g1 was up-regulated at three stages (1 DPI, 4 DPI and 12DPI). About 20% ROS-related genes up-regulated at 12 DPI, which suggested that at later stage, most of the ROS-related genes took part in the oxidative damage response upon *C*. *destructans* induction.

Ginsenosides are the major pharmacological component of *P*. *ginseng*, thus the genes involved in the ginsenoside biosynthetic pathway have been widely identified [31–34]. In total, we identified 248 unigenes involved in ginsenoside biosynthetic pathway, in which CYP450 was the biggest family that included 185 unigenes. There were 20 up-regulated CYP450s and 10 down-regulated CYP450s upon *C*. *destructans* infection. The other differentially expressed unigenes involved in ginsenoside biosynthetic pathway included four UDP-glycosyltransferases (UGT), four Acetyl-CoA acetyltransferase (AACT) and one squalene/phytoene synthase (SS). Most of the unigenes in the ginsenoside biosynthetic pathway were up-regulated upon *C*. *destructans* infection. In total, there were 4 up-regulated UGTs. Among them, c32203_g1 and c56056_g1 were up-regulated at 0.25 DPI and 0.5 DPI, respectively (S2 Fig and S7 Fig). C47595_g2 was up-regulated at both 0.5 DPI and 7 DPI. C57632_g1 was up-regulated at both 0.5 DPI and 12 DPI. At 12 DPI, four AACT (c50541_g1, c54663_g1, c59324_g1, c66211_g1) were up-regulated. Only one SS (c65219_g3) was down-regulated at both 0.5 DPI and 7 DPI. Above results suggested that ginsenoside biosynthetic pathway was affected upon *C*. *destructans* induction.

## Discussion

*De novo* transcriptome assembly of the RNA-Seq reads into full transcriptomes and the calculation of the expression level can be conducted for species with unknown genomes [13]. To date, several transcriptomes of *P*. *ginseng* focusing on ginsenoside biosynthetic genes have been reported [31–34]. However, global-transcriptome studies of *C*. *destructans* infection in *P*. *ginseng* remain lacking. The current study investigated *C*. *destructans* infection in *P*. *ginseng* and used high-throughput RNA-Seq to characterize the transcriptome profile of *P*. *ginseng* after *C*. *destructans* infection at different time points. The transcriptome upon *C*. *destructans* infection extended the transcriptome resources of *P*. *ginseng*.

**Fig 9:**
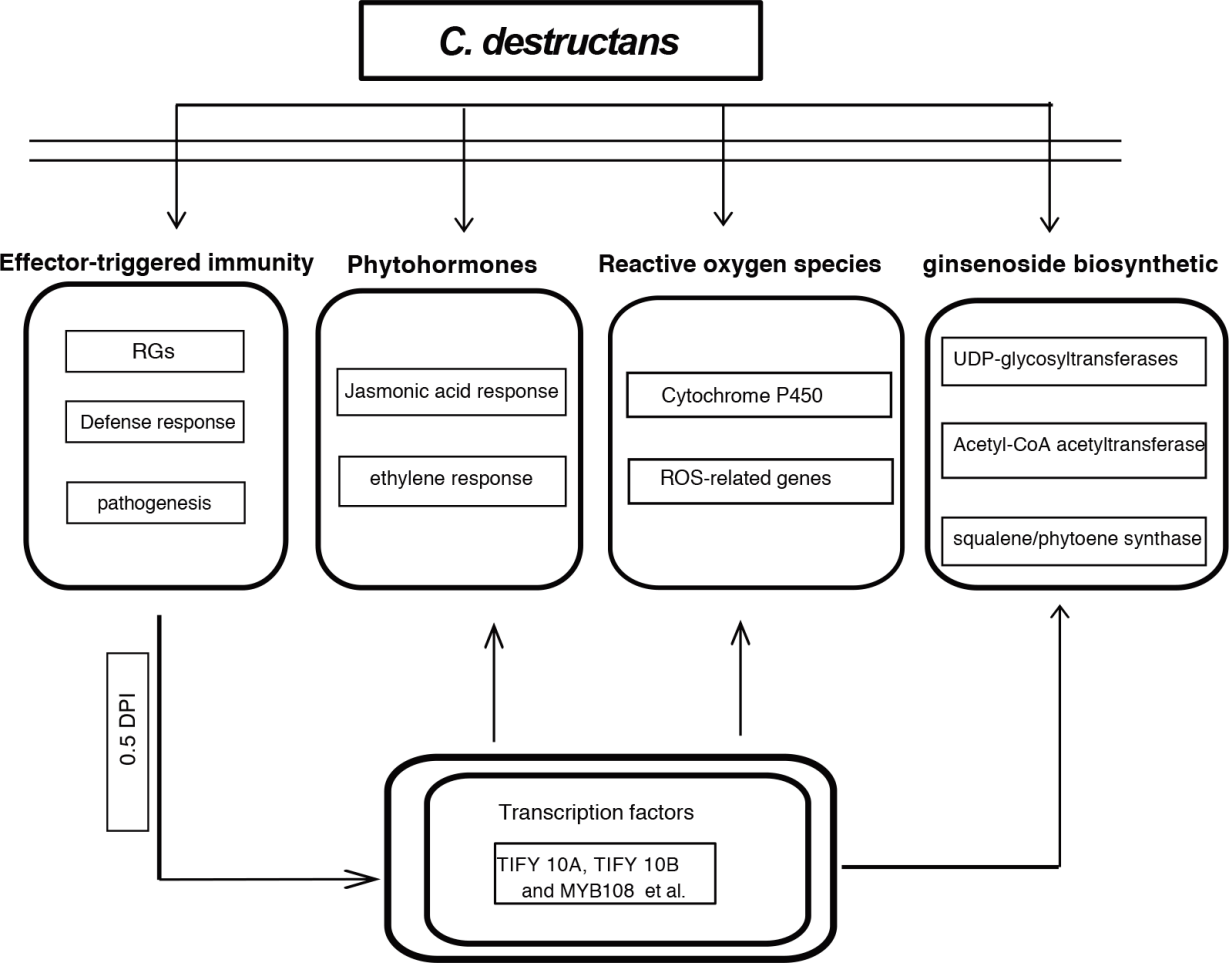
Model of *C*. *destructans* infection in the effector-triggered immunity, phytohormones, reactive oxygen species and ginsenoside biosynthetic pathway.

The induced response of *P*. *ginseng* to *C*. *destructans* infection was characterized to reveal defense-related genes involved in the plant-pathogen interaction. A total of 3,839 and 568 unigenes were up-regulated and down-regulated, respectively. Furthermore, pathogen response unigenes were up-regulated at 0.5 DPI. *P*. *ginseng* evolved an innate and inducible resistance or endurance to phytopathogens, pests, extreme environment, and other adverse factors. In the present study, RGs were dramatically induced by *C*. *destructans* at 0.5 DPI. Pathogen effector proteins from *C*. *destructans* can activate cytoplasmically localized R proteins during disease progression. These RGs might produce effector-triggered immunity (ETI) through gene-for-gene interaction (Fig 9). The DPI of 0.5 might be the essential point for ETI before *P*. *ginseng* obtained basal defenses through a phosphorylation cascade. After the immunity signal passed into nucleus, many defense related TFs (TIFY 10A, TIFY 10B and MYB108 et al) were activated. These transcript factors might further affected phytohormones, reactive oxygen species and ginsenoside biosynthetic pathway (Fig 9). *C*. *destructans* induction had effect on phytohormones, such as jasmonic acide and ethylene response. At the same time ROS-related genes might protect *P*. *ginseng* against invasion of *C*. *destructans* (Fig 9). Moreover, unigenes involved in ginsenoside biosynthetic might also play important roles in the response to *C*. *destructans* infection.

This study is the first to report on defense-related DEGs that potentially improve the resistance of *P*. *ginseng* against *C*. *destructans*-caused root rot disease. The results of this study deepen our understanding of pathogen–plant interactions. Furthermore, these candidate pathogen-response unigenes may be used to increase the resistance or tolerance of *P*. *ginseng* to root rot disease via transformation strategy. Most importantly, these canditated *C*. *destructans* response genes at 0.5 DPI were identified as essential genes for ETI, which could be used as markers for the early detection of *C*. *destructans* infection.

## Supporting Information

S1 Fig Bar plot of randomly selected DEGs. The y-axes represent normalized FPKM and the x-axes represent different stages after *C*. *destructans* infection.
(TIF)

S2 Fig Validation of DEGs from reactive oxygen species and ginsenoside biosynthetic pathway through qRT-PCR. Fold changes of the transcript levels at different time points are shown. The average expression level at 0 DPI was set to 1. Error bars represent standard error from three independent experimental replicates.
(TIF)

S3 Fig Bar plot of six representative DEGs for defense-response genes. The y-axes represent normalized FPKM and the x-axes represent different stages after *C*. *destructans* infection.
(TIF)

S4 Fig Bar plot of transcript factors and PRP unigenes. The y-axes represent normalized FPKM and the x-axes represent different stages after *C*. *destructans* infection.
(TIF)

S5 Fig Fold change of 8 DEGs for RGs. Unigenes with FPKM = 0 in uninfected library are not shown in this figure.
(TIF)

S6 Fig Bar plot of six representative DEGs for reactive oxygen species. The y-axes represent normalized FPKM and the x-axes represent different stages after *C*. *destructans* infection.
(TIF)

S7 Fig Bar plot of 9 DEGs for ginsenoside biosynthetic pathway. The y-axes represent normalized FPKM and the x-axes represent different stages after *C*. *destructans* infection.
(TIF)

S1 Table Primers used for qRT-PCR validation. (DOC)

S2 Table List of all differentially expressed genes with GO annotation. (XLS)

S3 Table List of all identified RGs. (XLS)
