# Supplementary figures and images for: Time-Course Transcriptome Analysis Reveals Resistance Genes of Panax ginseng Induced by Cylindrocarpon destructans Infection Using RNA-Seq
Source: PLoS One. 2016 Feb 18;11(2):e0149408. doi: 10.1371/journal.pone.0149408 (PMC4758610; doi:10.1371/journal.pone.0149408)

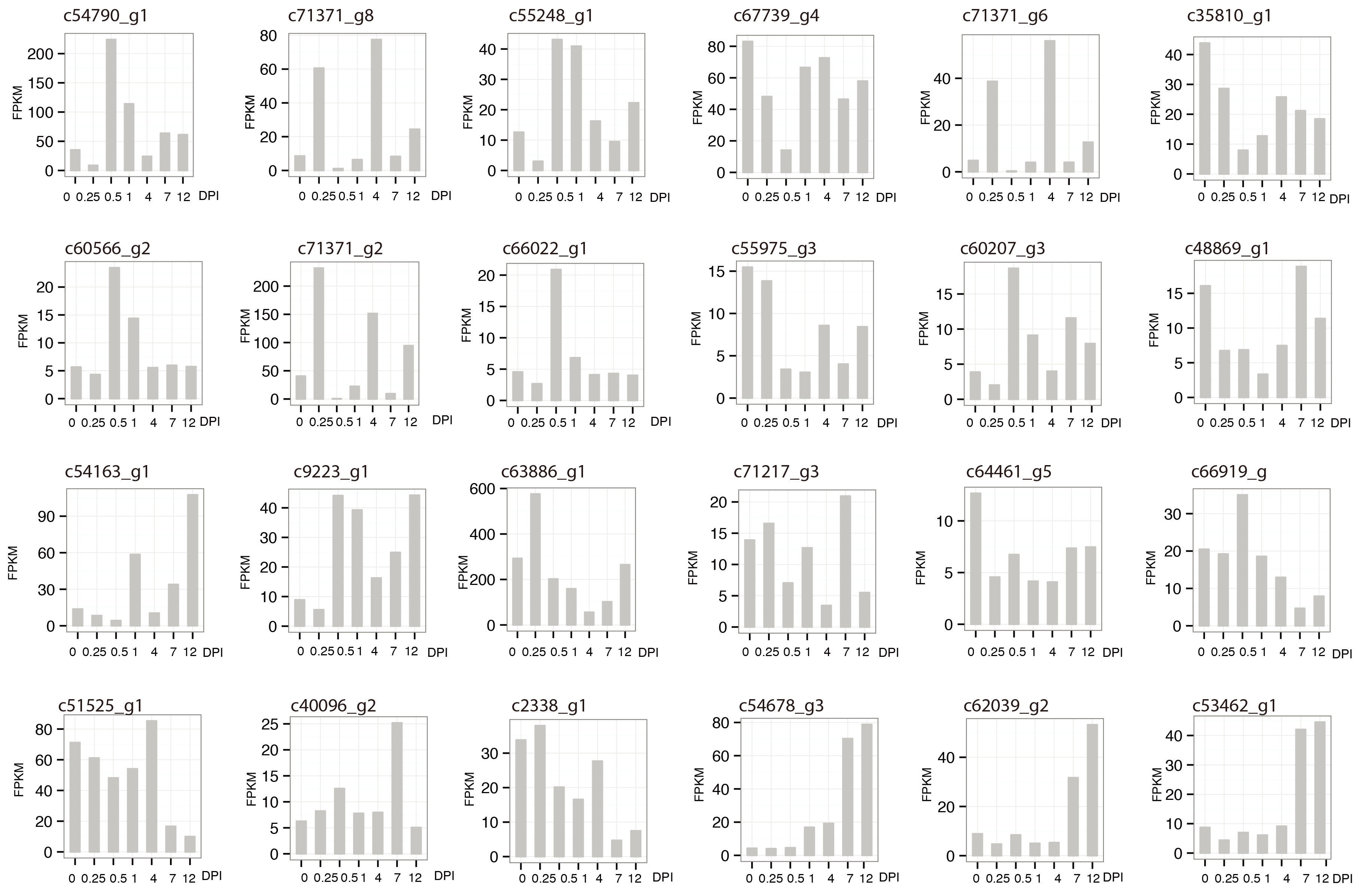

Supplement: S1 Fig — The y-axes represent normalized FPKM and the x-axes represent different stages after C. destructans infection. (TIF) [file pone.0149408.s001.tif]

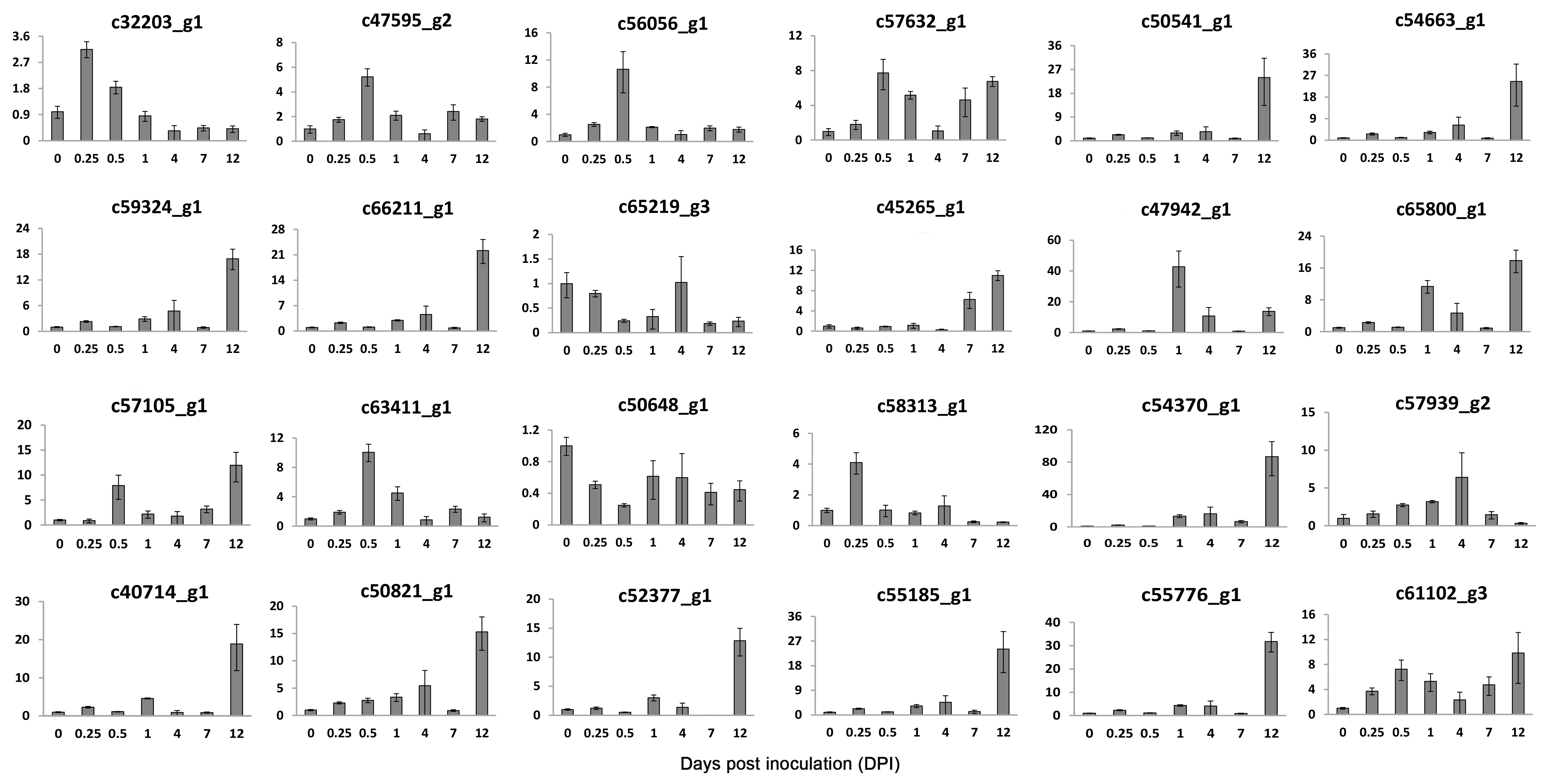

Supplement: S2 Fig — Fold changes of the transcript levels at different time points are shown. The average expression level at 0 DPI was set to 1. Error bars represent standard error from three independent experimental replicates. (TIF) [file pone.0149408.s002.tif]

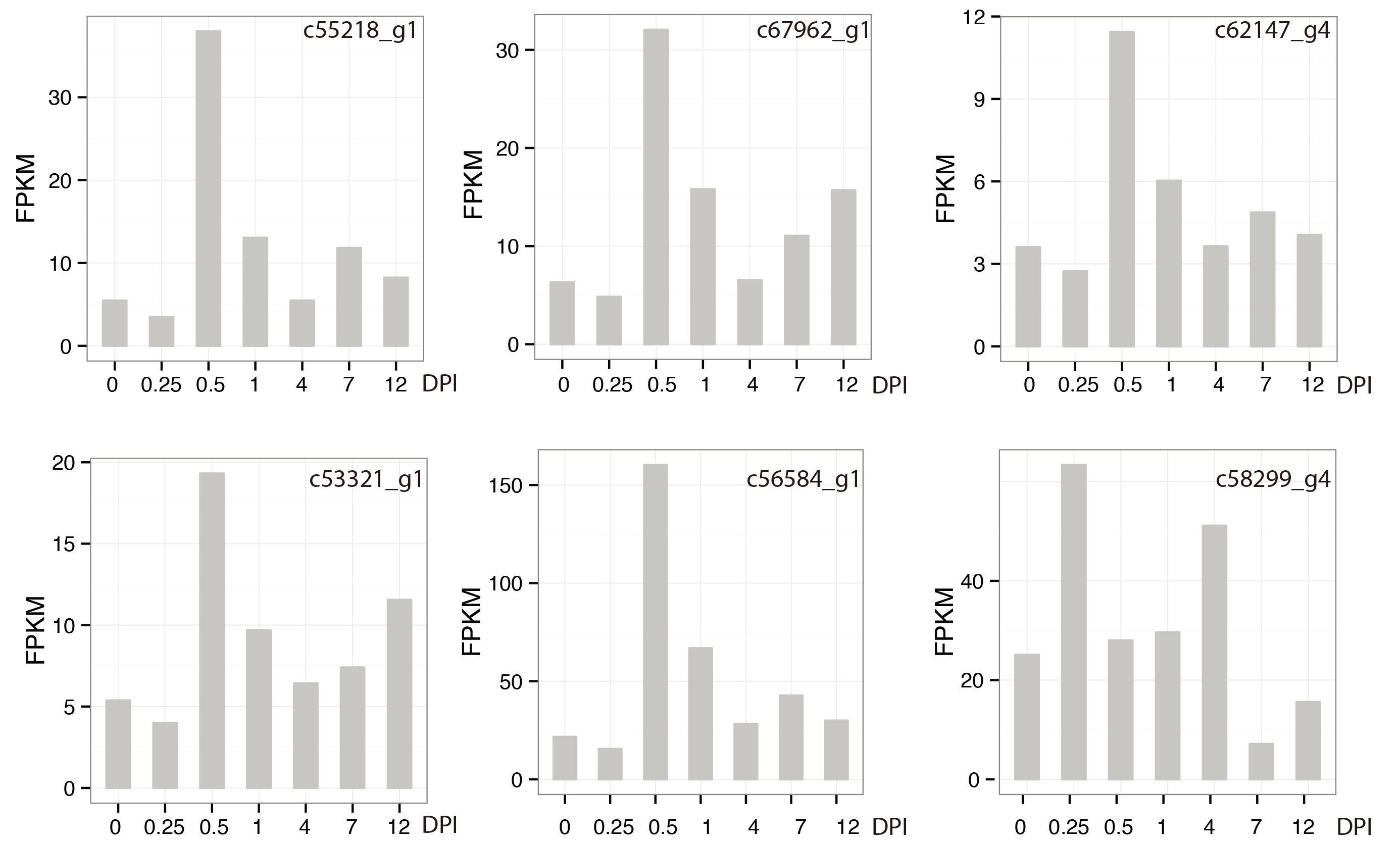

Supplement: S3 Fig — The y-axes represent normalized FPKM and the x-axes represent different stages after C. destructans infection. (TIF) [file pone.0149408.s003.tif]

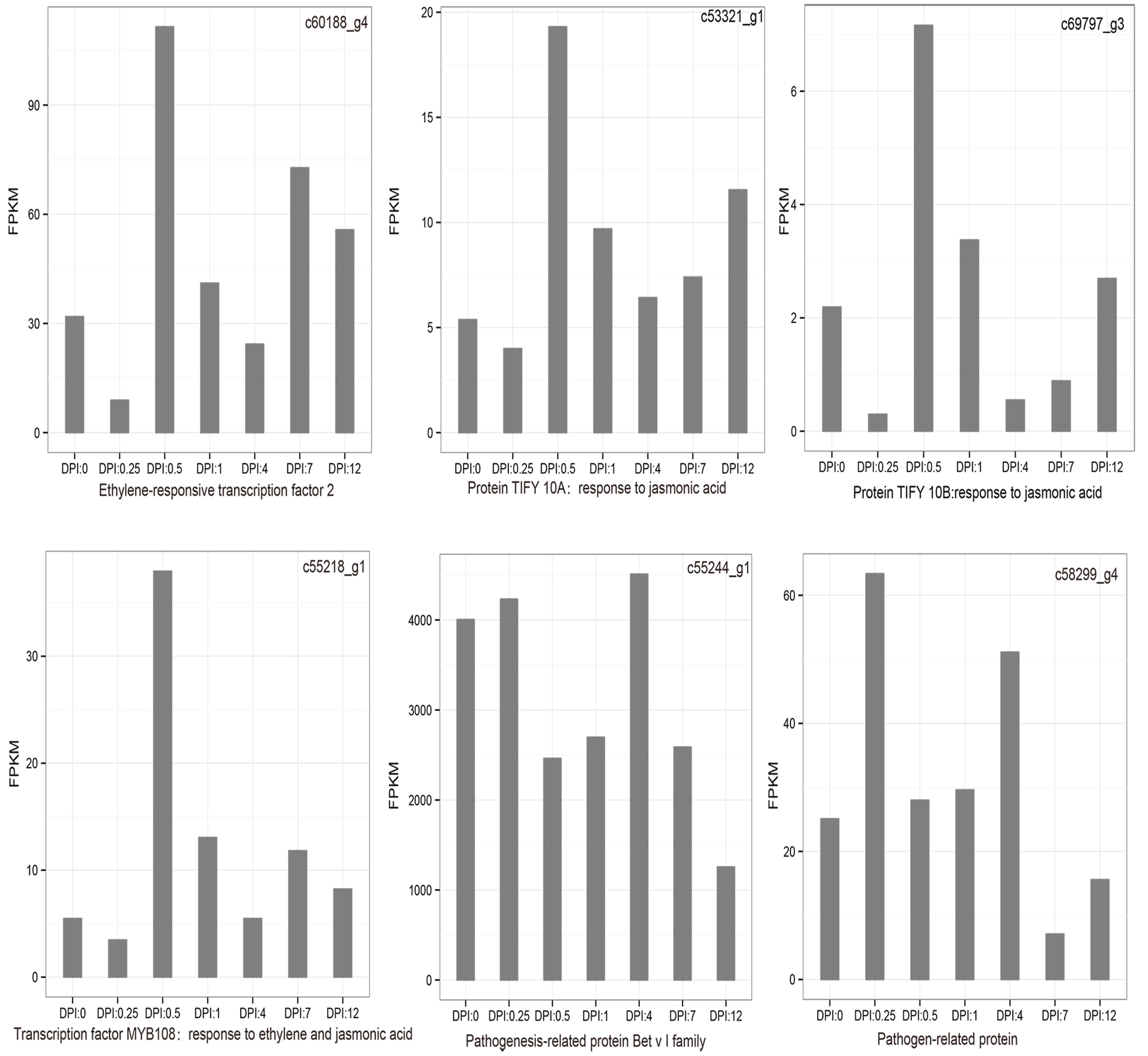

Supplement: S4 Fig — The y-axes represent normalized FPKM and the x-axes represent different stages after C. destructans infection. (TIF) [file pone.0149408.s004.tif]

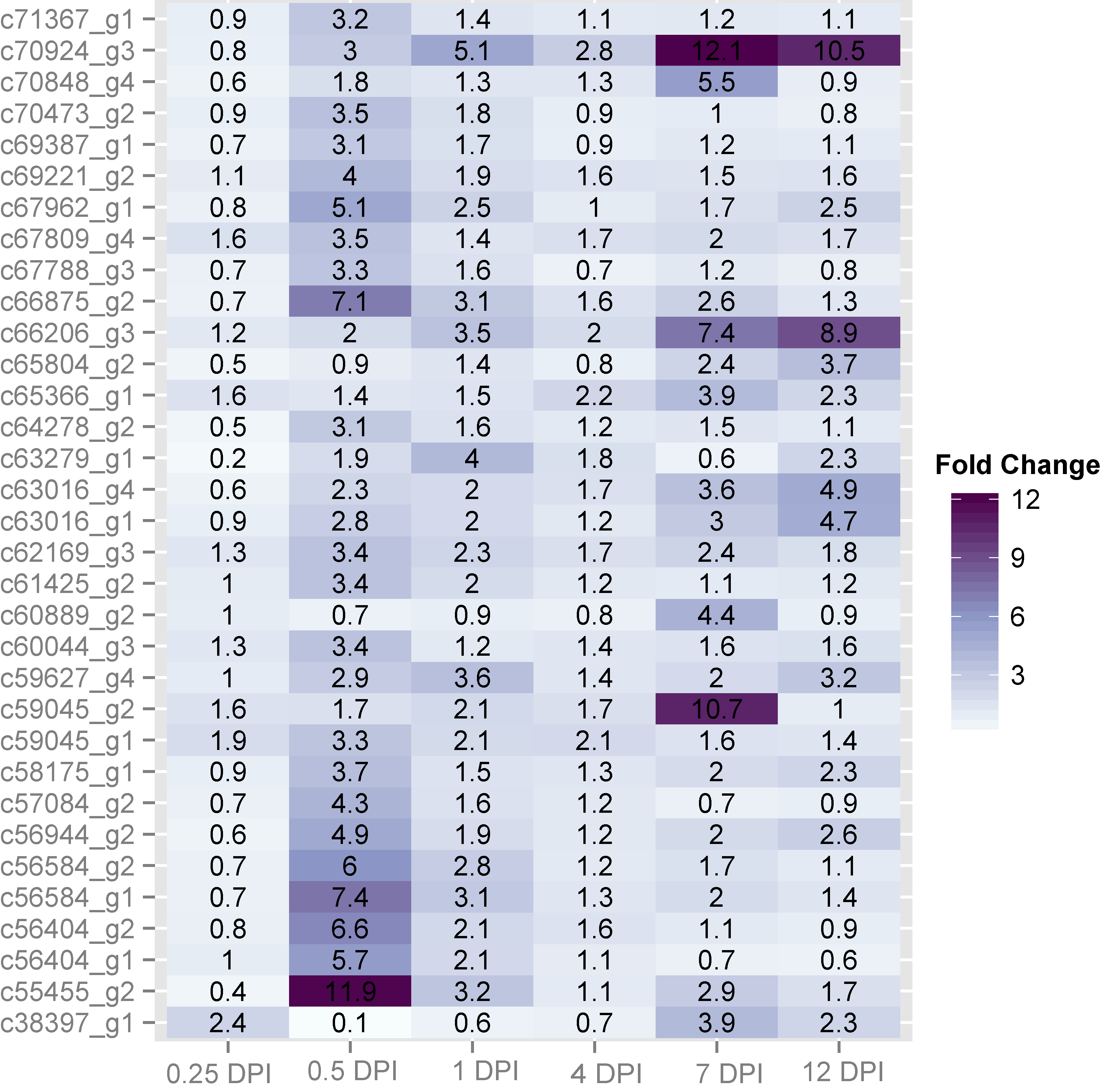

Supplement: S5 Fig — Unigenes with FPKM = 0 in uninfected library are not shown in this figure. (TIF) [file pone.0149408.s005.tif]

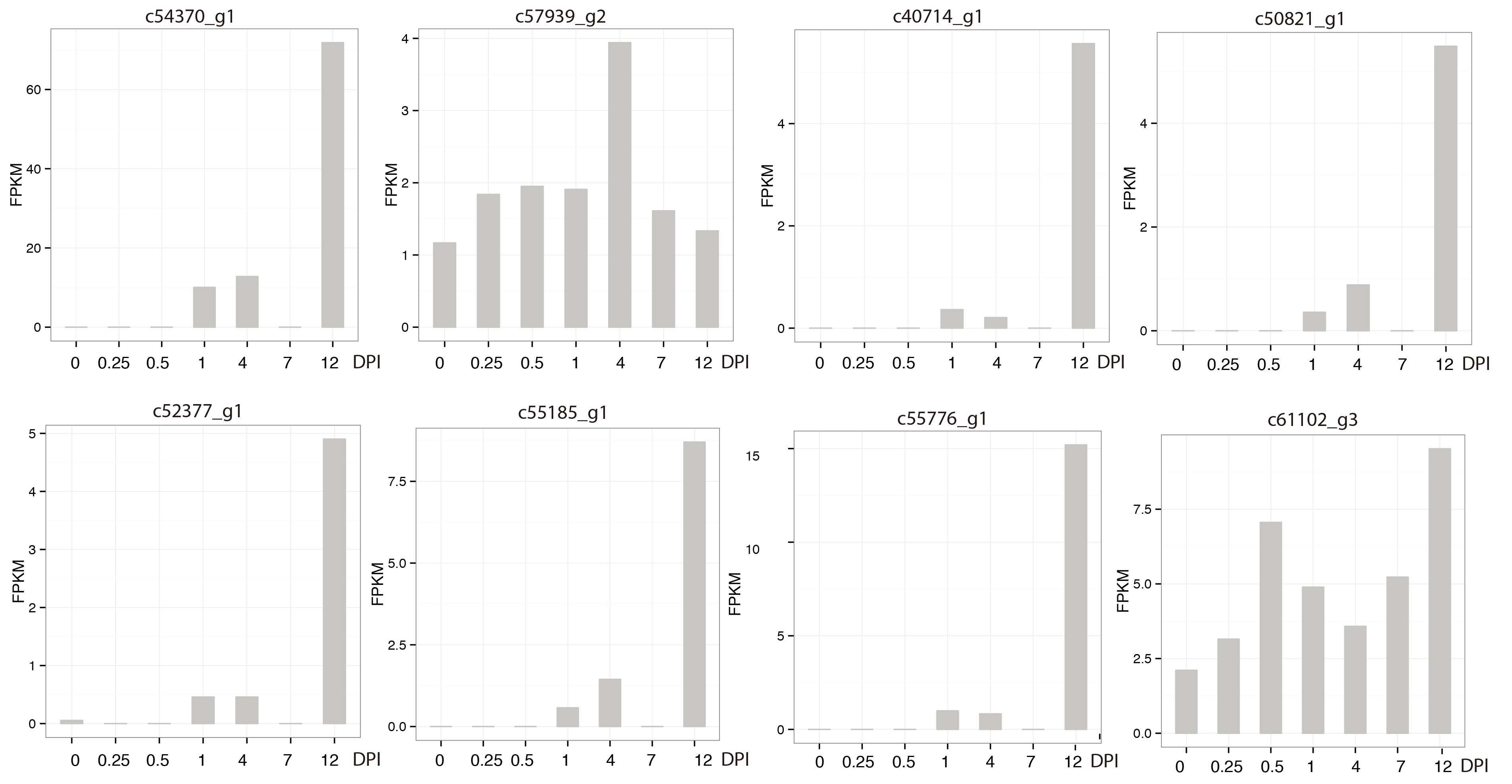

Supplement: S6 Fig — The y-axes represent normalized FPKM and the x-axes represent different stages after C. destructans infection. (TIF) [file pone.0149408.s006.tif]

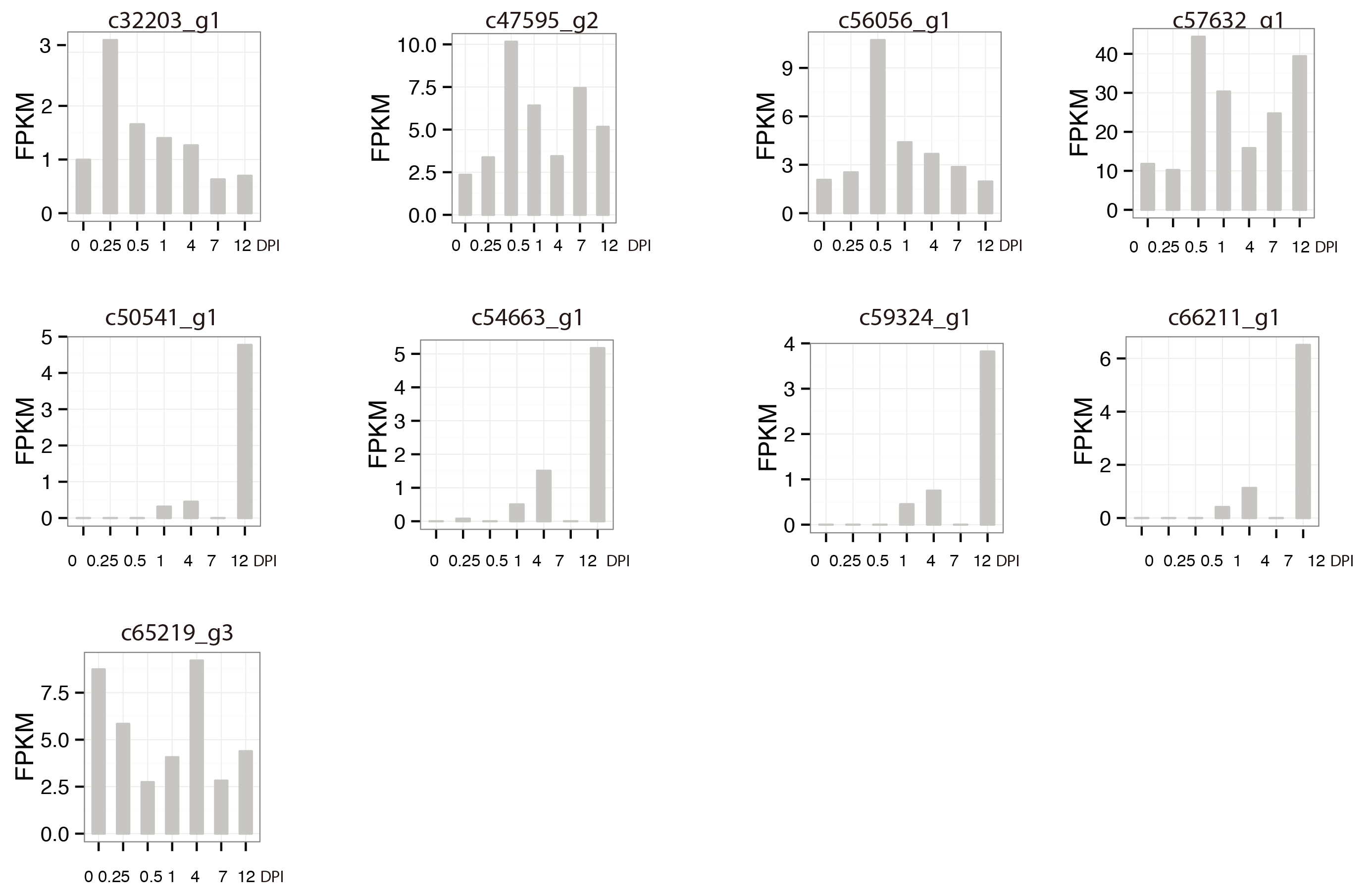

Supplement: S7 Fig — The y-axes represent normalized FPKM and the x-axes represent different stages after C. destructans infection. (TIF) [file pone.0149408.s007.tif]
