# Supplementary material for: Time-Course Transcriptome Analysis Reveals Resistance Genes of Panax ginseng Induced by Cylindrocarpon destructans Infection Using RNA-Seq
Source: PLoS One. 2016 Feb 18;11(2):e0149408. doi: 10.1371/journal.pone.0149408 (PMC4758610; doi:10.1371/journal.pone.0149408)
Supplement: S1 Table — (DOC) [file pone.0149408.s008.doc]

**Table S1. Primers used for qRT-PCR**

| **Genes** | **primers** | **sequences** |
| --- | --- | --- |
| c55218_g1 | Forward | AAAGATTGCTCGACACCTGC |
|  | Reverse | TCATTTCCTCAAACGCGGAG |
| c53321_g1 | Forward | GTCGGGCCAAATATCCACAC |
|  | Reverse | TTTTAGGCTCCATTGCTGCC |
| c62147_g4 | Forward | CCACATATGAGCAAACCCGG |
|  | Reverse | AGACGTCCTAGTTTTGGGCA |
| c58299_g4 | Forward | GGAAAGGTGCGACAATGGAA |
|  | Reverse | AAACGCTGTGGTGAAAACGA |
| c56584_g1 | Forward | GGCACCTTACTCATGTCCCT |
|  | Reverse | CGCTTAAACCCACAGCTCTG |
| c67962_g1 | Forward | GTTACCAGTTAGCATGGCGG |
|  | Reverse | ATCTACCCAGTTCCTTGGCC |
| c54790_g1 | Forward | CCGAAGCCCAGAAACTTGTC |
|  | Reverse | GGGAAAGAAAACCTCAGCCC |
| c55248_g1 | Forward | ATTCATGGCCTACGACCCAT |
|  | Reverse | TCGCCACAAACTACATCCCT |
| c71371_g8 | Forward | GTCAGCGGAAGAAAAGGCAG |
|  | Reverse | TTGACTAAACCACCCAGCCT |
| c71371_g6 | Forward | CTGAGCGTCAAAACCGGAAA |
|  | Reverse | AAATTGACGAAGACCTGGCG |
| c71371_g2 | Forward | GGTTGTATGACACGGAAGGC |
|  | Reverse | TTCTCGGTACTGCTCCCCTA |
| c67739_g4 | Forward | CAGCAAGGATCCCAGAGACC |
|  | Reverse | TCCCTTTCATGCGGTTCTCT |
| c35810_g1 | Forward | TTTGAGTCCGAACAGAGCCT |
|  | Reverse | TCCCAACTCCCACTCACAAA |
| c60566_g2 | Forward | GGCGGAGGTGATGGTAGTAA |
|  | Reverse | AGATCAACTCCCATGTGGCA |
| c66022_g1 | Forward | GTAACATTGACACGAGCGGG |
|  | Reverse | AAGAACTTGGCCTTGCTCAG |
| c60207_g3 | Forward | ACCAACCCTACAGAAGCCTC |
|  | Reverse | AACATTGTGCCTGCCCAAAT |
| c55975_g3 | Forward | ACCTCAGCAACAACAGCAAG |
|  | Reverse | TATGGAGCTAACTGTGGCCC |
| c48869_g1 | Forward | TCTATCGAGCAGCCAAACCT |
|  | Reverse | TGAGGCAGTAGAAGGAGCAA |
| c54163_g1 | Forward | AGGAGGTAAATGGCTCCTAACA |
|  | Reverse | ACTTGGTTCTCACTGGACGA |
| c9223_g1 | Forward | GTGCCTGTTTCGTCACACAA |
|  | Reverse | CCCACTTGCCACACTTTGAA |
| c63886_g1 | Forward | TGTAGCCGTACCATTTGTGC |
|  | Reverse | TGAACCAACAAGCAACAAGGT |
| c71217_g3 | Forward | CTGCCTTCCTTCCACAGAGA |
|  | Reverse | AGGGATTTGGCTCTGTTTGC |
| c64461_g5 | Forward | ATCCGGGCTGCTGAAGAAG |
|  | Reverse | ATTCGCGGTATCTCCCCAAT |
| c54678_g3 | Forward | TTGATCAGCCTGGCCAGATA |
|  | Reverse | CAAGGCATGCTCAGGGAATC |
| c66919_g1 | Forward | GACATTACCTCCTTTGCCGG |
|  | Reverse | TCAAGGGCTAGTGGTGGATG |
| c51525_g1 | Forward | CCGTAATCTTCAAGGGCACG |
|  | Reverse | GTTCCCCAAGCCAACACAAG |
| c40096_g2 | Forward | AAAGCCGCAGATTTGGTACG |
|  | Reverse | TTACGACGTTGAAGGCAGAC |
| c2338_g1 | Forward | TGGTTGGTCGGCATTTTGTT |
|  | Reverse | TGCAGTGGATTTTGTGGTGG |
| c62039_g2 | Forward | CTGAATGCGCTGATCTTCCC |
|  | Reverse | ACCAGAACCCGTTACAACCT |
| c53462_g1 | Forward | GCAATGGAACCGCATTACCT |
|  | Reverse | CCCGGTTCGAGGAATACAGA |
| c54370_g1 | Forward | CATCATTTGGCGTCTGTCCC |
|  | Reverse | GTTGCACACTTTCCACCGAA |
| c57939_g2 | Forward | GATGCCAAAGATGACCCACC |
|  | Reverse | TTGCAAGCAATGTGAGCAGT |
| c40714_g1 | Forward | GCGAACGGATTTAGCACTGT |
|  | Reverse | GCATTTCCAGTTGTCGACGA |
| c50821_g1 | Forward | TCAGCGACATGTCTCATCGA |
|  | Reverse | CGTTCATTGTGATTTGCCGC |
| c52377_g1 | Forward | AACATTGCTATCCCGTTCGC |
|  | Reverse | GCTGGCATCTTTCGGACAAT |
| c55185_g1 | Forward | GCAGTTGACTTCGGGTGATG |
|  | Reverse | TCCATTCCAGCGATGTCAGT |
| c55776_g1 | Forward | TCTTGCGACCGACTATCACA |
|  | Reverse | AAGACTGCACTTTTGGCGG |
| c61102_g3 | Forward | CTCTGGATGGAGCGGTGATA |
|  | Reverse | GTGGTTTATCTGATGGGCGC |
| c32203_g1 | Forward | CTGTTGGTGGGTTTCTCAGC |
|  | Reverse | ACATCTCTCCCACACACCTG |
| c56056_g1 | Forward | ACTCATTCCATCCACCAGCA |
|  | Reverse | CGATCGAATTCTAGCGGCAG |
| c57632_g1 | Forward | ATTTCATCCATCCAACCGCG |
|  | Reverse | GCTTCTTTTGTGTCGAGCCA |
| c47595_g2 | Forward | GTCGGTCAGAGATTTCGTGC |
|  | Reverse | CCGCCATCTTCATCCACAAG |
| c50541_g1 | Forward | TTGATTGATCGCGGTGACAC |
|  | Reverse | TTATCCGCCAAGCCTCTCAA |
| c54663_g1 | Forward | CCTATGGCTCTGACTGCTGA |
|  | Reverse | TGAAGGGAGTGATTTCGGCT |
| c59324_g1 | Forward | TTGCTGGAGAAGAATGGGGT |
|  | Reverse | CCGCCATGAATGTTGACCTT |
| c66211_g1 | Forward | TCGTTGCCAATCTGAAAGCC |
|  | Reverse | TGGACAATGATCCACCCCAA |
| c65219_g3 | Forward | CAGGCTACTACGGAGAGTGT |
|  | Reverse | CTTGCGCCAGTTCATCTTGT |
| c45265_g1 | Forward | AAGATTGTGCCCCGGAATTG |
|  | Reverse | AGTCCAACATCCTCGGACAT |
| c47942_g1 | Forward | ACCGGTCTTCACTACGATCC |
|  | Reverse | AGGCCCATCTCCAAATTCCA |
| c65800_g1 | Forward | TGGTGCATTGACGAGACTCT |
|  | Reverse | CCGGAATGGTAATTGGCGTT |
| c57105_g1 | Forward | AAGCAAGGGAGGACATTCGA |
|  | Reverse | CTCTCCGGTCTGAACTCGTT |
| c63411_g1 | Forward | AGGGTGGTGAGCTTGATGAT |
|  | Reverse | CTTTGGGATGGCAAGTAGTGG |
| c50648_g1 | Forward | GCAATATCTCCACGCTGCAA |
|  | Reverse | GCATTATACGTCGCGAACCA |
| c58313_g1 | Forward | CGAGTTGAATGTGCAGCCTT |
|  | Reverse | GCACAACTTGGAGGGTAAGC |
